# The PHD and Chromo Domains Regulate the ATPase Activity of the Human Chromatin Remodeler CHD4

**DOI:** 10.1016/j.jmb.2012.04.031

**Published:** 2012-09-07

**Authors:** Aleksandra A. Watson, Pravin Mahajan, Haydyn D.T. Mertens, Michael J. Deery, Wenchao Zhang, Peter Pham, Xiuxia Du, Till Bartke, Wei Zhang, Christian Edlich, Georgina Berridge, Yun Chen, Nicola A. Burgess-Brown, Tony Kouzarides, Nicola Wiechens, Tom Owen-Hughes, Dmitri I. Svergun, Opher Gileadi, Ernest D. Laue

**Affiliations:** 1 Department of Biochemistry, University of Cambridge, Cambridge CB2 1GA, UK; 2 The Structural Genomics Consortium, University of Oxford, ORCRB, Roosevelt Drive, Oxford OX3 7DQ, UK; 3 European Molecular Biology Laboratory-Hamburg Outstation, c/o DESY, Notkestrasse 85, Hamburg, Germany; 4 Cambridge Centre for Proteomics, Department of Biochemistry and Cambridge Systems Biology Centre, University of Cambridge, Cambridge, UK; 5 Department of Bioinformatics and Genomics, University of North Carolina at Charlotte, Charlotte, NC 28023, USA; 6 The Gurdon Institute, Department of Pathology, Cambridge, UK; 7 Wellcome Trust Centre for Gene Regulation and Expression, College of Life Sciences, University of Dundee, Dundee DD1 5EH, UK

**Keywords:** CHD, chromo domain helicase DNA binding, NuRD, nucleosome remodeling and deacetylase, PHD, plant homeodomain, SAXS, small-angle X-ray scattering, LC–MS/MS, liquid chromatography–tandem mass spectrometry, DUF, domain of unknown function, TEV, tobacco etch virus, HRP, horseradish peroxidase, BSA, bovine serum albumin, Bistris, 2-[bis(2-hydroxyethyl)amino]-2-(hydroxymethyl)propane-1,3-diol, NuRD complex, chromatin remodeling, chromo domain helicase DNA-binding protein 4, histone, transcriptional regulation

## Abstract

The NuRD (nucleosome remodeling and deacetylase) complex serves as a crucial epigenetic regulator of cell differentiation, proliferation, and hematopoietic development by coupling the deacetylation and demethylation of histones, nucleosome mobilization, and the recruitment of transcription factors. The core nucleosome remodeling function of the mammalian NuRD complex is executed by the helicase-domain-containing ATPase CHD4 (Mi-2β) subunit, which also contains N-terminal plant homeodomain (PHD) and chromo domains. The mode of regulation of chromatin remodeling by CHD4 is not well understood, nor is the role of its PHD and chromo domains. Here, we use small-angle X-ray scattering, nucleosome binding ATPase and remodeling assays, limited proteolysis, cross-linking, and tandem mass spectrometry to propose a three-dimensional structural model describing the overall shape and domain interactions of CHD4 and discuss the relevance of these for regulating the remodeling of chromatin by the NuRD complex.

## Graphical Abstract

**Figure:**
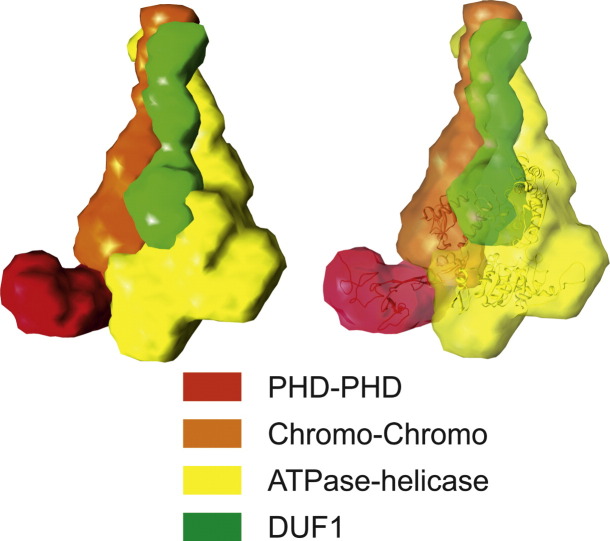

**Highlights:**

► The ATPase CHD4 mediates nucleosome remodeling by the NuRD complex. ► We present a three-dimensional small-angle X-ray scattering model of CHD4 and define its interdomain interactions. ► Cross-linking and limited proteolysis studies validate our model. ► Functional and binding assays suggest a regulatory role for the PHD and chromo domains.

## Introduction

The nucleosome is the basic element of chromatin and is composed of approximately 147 bp of DNA tightly wrapped around a histone octamer comprising two copies of each of the core histones H2A, H2B, H3, and H4. Chromosomal packing varies widely and depends on the intrinsic DNA-binding propensity of core particles, the presence of histone variants, and a plethora of posttranslational modifications together with their interacting proteins, as well as proteins that remodel chromosome structure. The resulting variation in the accessibility of the DNA plays an essential role in the processes of transcription, DNA replication, and repair. Therefore, the chromatin structure is tightly controlled both by remodeling/rearrangement of histones and by the addition/removal of specific posttranslational modifications, mediating the recruitment of particular complexes to specific genes.^1^ These activities are coupled largely through the action of ATP-dependent chromatin remodeling complexes.^1^ The sequence identity of the ATPase component can be used to classify these complexes into the SWI/SNF, ISWI, INO80, and CHD (chromo domain helicase DNA binding) families.^1,2^ The CHD family is so named for its tandem chromo domains located towards the N-terminus of each protein and for its central SNF2-like ATPase motif.^2,3^ The CHD family can be divided into three groups (CHD1–2, CHD3–5, and CHD6–9) according to the presence or absence of additional domains. It can be further divided into 24 subfamilies, based on sequence differences within the domains.^3,4^ The CHD3–5 subfamily includes the proteins CHD3 and CHD4 (also known as Mi-2α and Mi-2β, respectively).^3^ These proteins contain paired N-terminal PHD (plant homeodomain) domains that are absent from the CHD1–2 subfamily and are implicated in the regulation of transcriptional processes.^3,5–8^ CHD3–5 subfamily members also appear to lack the C-terminal HAND-SANT-SLIDE motif that has recently been shown to mediate DNA binding in CHD1 and in members of the CHD6–9 subfamily.^9^ CHD4 was initially identified as a dermatomyositis-specific autoantigen and has now been shown to execute the chromatin remodeling activity of the NuRD (nucleosome remodeling and deacetylase) complex.^10,11^ The NuRD complex links multiple transcriptional regulatory processes, including histone deacetylation, histone demethylation, nucleosome mobilization, and regulatory protein recruitment and is a critical epigenetic regulator of hematopoietic development, differentiation, and cancer.^10,12,13^ Alongside its nucleosome remodeling ATPase subunit (CHD3 or CHD4), the NuRD complex also contains the histone deacetylases HDAC1 and HDAC2, the histone chaperones RbAp46 and RbAp48, the methyl binding domain proteins MBD2, or more commonly MBD3, the metastasis tumor antigen subunits 1/2/3, and the GATA-type zinc finger transcription factors GATA2a and b.^10^ The potential tumor suppressor protein DOC1 (deleted in oral cancer) has also been identified as a NuRD complex component.^14,15^ The CHD4 ATPase is an active nucleosome remodeler in the absence of the other NuRD complex components and is capable of both stimulating the sliding of individual nucleosomes along sections of DNA and perturbing the association of histone proteins with DNA.^11^ However, the precise mechanism by which the enzymatic function of CHD4 is regulated and the potential roles of its PHD and chromo domains in this regard have not yet been elucidated. Here, we present a model for CHD4 domain interactions and discuss the relevance of these for regulating chromatin remodeling activity.

## Results and Discussion

### Expression, purification, and characterization of human CHD4 constructs

**Fig. 1:**
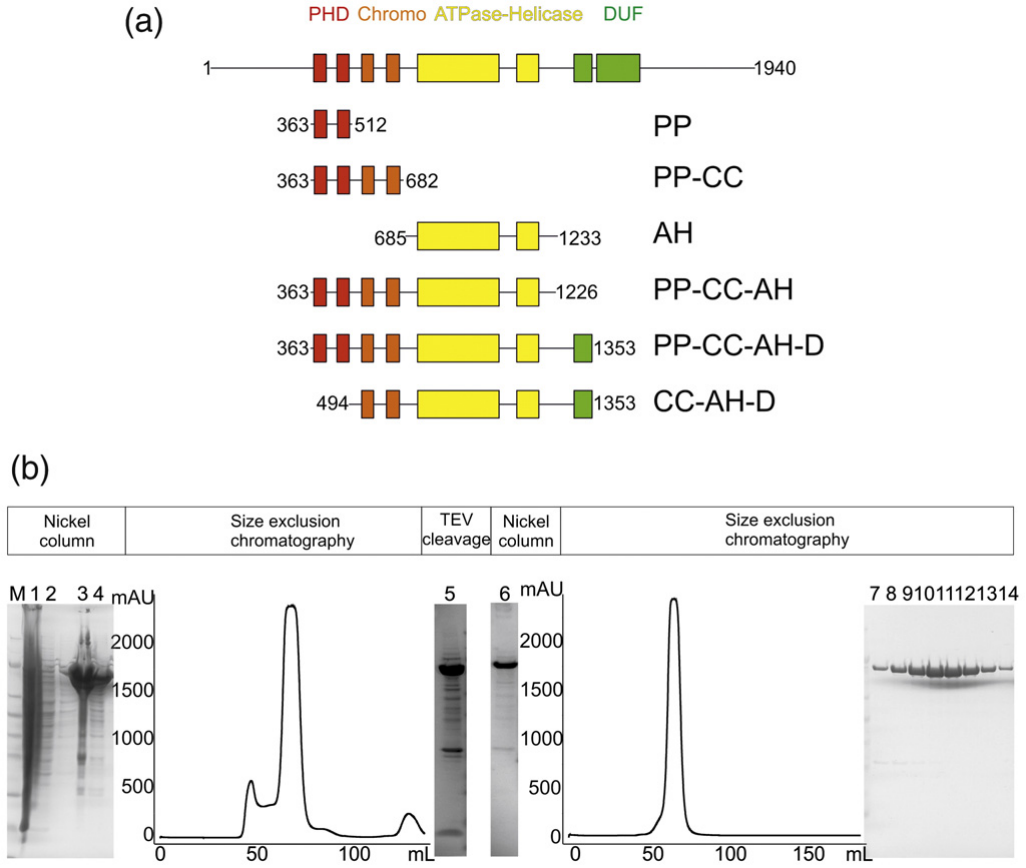
Recombinant CHD4 protein production and characterization. (a) Human CHD4 protein constructs. PHD (P) domains are colored red, chromo (C) domains are colored orange, the ATPase-helicase (AH) domain is colored yellow, and the domains of unknown function (D) are colored green. (b) Purification strategy for CHD4 constructs. Here, SDS-PAGE gels are presented following the nickel affinity purification, TEV protease cleavage, and size-exclusion chromatography steps applied to construct CC-AH-D. Similar strategies were followed for the other CHD4 constructs. Lane 1, total lysate; lanes 2–4, the elutions from nickel beads using buffer supplemented with 10, 30, and 300 mM imidazole, respectively; lane 5, TEV-cleaved protein; lane 6, flow-through following reapplication of TEV-cleaved protein to a second nickel resin column; lanes 7–14, fractions from a peak with an elution volume corresponding to the predicted molecular mass of CC-AH-D, eluted from HiLoad Superdex 200 16/60 Prep Grade filtration column. Protein samples were resolved on a NuPAGE 4–12% Bistris gel stained with Coomassie Brilliant Blue stain.

Recombinant human CHD4 proteins containing either an N-terminal hexahistidine or a C-terminal decahistidine tag were expressed in Sf-9 insect cells and were purified on a nickel affinity column followed by size-exclusion chromatography (Fig. 1 and Supplementary Fig. 1). Tobacco etch virus (TEV) protease was used to detach the histidine tags, and the cleaved proteins were further purified using a second nickel column (to remove both the tag and the histidine-tagged TEV protease) followed by a second size-exclusion chromatography step (Fig. 1b and Supplementary Fig. 1). The following protein constructs were expressed and purified in this way (Fig. 1a): PP-CC-AH-D, PP-CC-AH, PP-CC, PP, CC-AH-D, and AH [where P = PHD domain, C = chromo domain, AH = ATPase-helicase domain, and D = domain of unknown function (DUF) 1]. The purity, molecular masses, and monodispersity of each of these constructs were assessed by SDS-PAGE, electrospray ionization time-of-flight mass spectrometry, and dynamic light scattering, respectively (data not shown).

### The PHD domains of CHD4 mediate its binding to nucleosomes

**Fig. 2:**
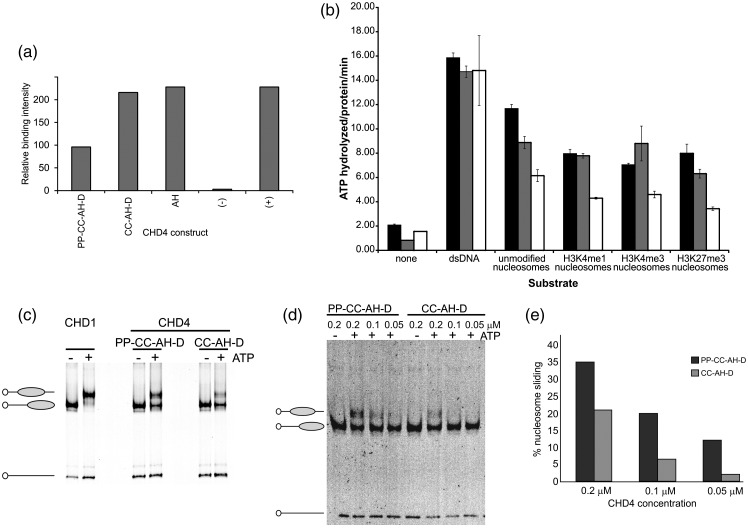
CHD4 nucleosome binding, sliding, and ATPase assays. (a) Representative quantifications of dot blots showing binding of unmodified nucleosomes by CHD4 constructs. The CHD4 proteins PP-CC-AH-D, CC-AH-D, and AH, alongside an unrelated negative control protein (−) and nucleosomes (+), were spotted onto a nitrocellulose membrane and incubated with unmodified biotinylated nucleosomes. Bound nucleosomes were detected with HRP-conjugated streptavidin. The spot intensities relative to background are plotted as a histogram. (b) ATPase assays were performed for constructs PP-CC-AH (black histogram), CC-AH-D (gray histogram), and AH (white histogram) in the presence or absence of the following substrates: double-stranded DNA (dsDNA) containing the 601 nucleosome positioning sequence; nucleosomes that were either unmodified, or mono- or trimethylated at lysine 4 of histone H3 (H3K4me1 or H3K4me3), or trimethylated at lysine 27 of histone H3 (H3K27me3). Mean activity is plotted as units of ATP hydrolyzed per protein per minute, and symmetric error bars are drawn with a length of 2 SD. The results of three experimental data sets are shown. (c) Nucleosomes (0.1 μM) assembled on a Cy3-labeled 0W47 DNA species containing a nucleosome positioning sequence were incubated with a 0.3 μM solution of either PP-CC-AH-D or CC-AH-D in the presence (+) or absence (−) of 1 mM ATP for 30 min at 37 °C. Samples were loaded onto a 5% 0.2× TBE native polyacrylamide gel before being scanned with a phosphoimager. Yeast CHD1 protein was used as a control. (d) Representative quantifications of nucleosome sliding experiments. 0W47 nucleosome sliding assays were repeated using a range of concentrations (0.05–0.2 μM) of CHD4 proteins PP-CC-AH-D and CC-AH-D for 1 h at 37 °C. Cy3-labeled (open circle), DNA (black line), and nucleosomes (gray ovals) are represented pictorially to the left of the gels in (c) and (d). (e) The percentages of nucleosome sliding in (d) at each concentration of PP-CCAH-D (dark gray) and CC-AH-D (light gray) are presented as histograms. Here, 100% represents the sum of the two nucleosomal bands in each assay, and each repositioned nucleosomal band is shown as a percentage of this sum.

CHD4 imparts to the NuRD complex the ability to translocate nucleosomes along DNA.^10^ The interaction of the PHD domains of CHD4 with the N-terminal tails of histone H3 has been previously reported, but the relative importance of these and other domains of CHD4 in mediating interactions with nucleosomes has not been characterized.^6,7^ We performed dot blots using horseradish peroxidase (HRP)-conjugated streptavidin to detect interactions between biotinylated nucleosomes and the CHD4 constructs PP-CC-AH-D, CC-AH-D, and AH. These experiments suggest that the presence of the PHD domains reduces the binding of CHD4 to free nucleosomes (compare the binding of the fragment PP-CC-AH-D with CC-AH-D and AH in Fig. 2a and Supplementary Fig. 3). These results imply a role for the PHD domains in modulating nucleosome interactions, and we therefore sought to investigate whether the presence of these motifs, and of the chromo domains, affects the ATPase/helicase function of CHD4.

### The ATPase and nucleosome remodeling activities of CHD4 are regulated by its PHD and chromo domains

To assess the influence of the PHD and chromo domains of CHD4 on its activity, we performed ATPase assays in the presence of DNA, nucleosomes, or buffer, using recombinant proteins comprising the ATPase-helicase domain alone (construct AH), or with the tandem chromo domains (construct CC-AH-D), or with both the tandem PHD and chromo domains (construct PP-CC-AH) added (Fig. 2b).

As in the context of the intact NuRD complex, the ATPase activity of CHD4 is stimulated both by nucleosomes and by the same DNA, which has not been assembled into nucleosomes (Fig. 2b).^11^ In the presence of double-stranded DNA, the CHD4 construct lacking both the PHD and chromo domains (AH) appears to be as efficient an ATPase as those constructs that contain these additional domains (Fig. 2b). This suggests that all three different protein constructs translocate along naked DNA equally efficiently, although it is not known whether ATP hydrolysis mediated by CHD4 is coupled to DNA translocation. Although it is possible that nucleosomes prevent the DNA from engaging efficiently with the enzyme, when presented with nucleosomes, the ATPase activity of all of the constructs was reduced, consistent with the nucleosomes acting as a barrier to translocation. Interestingly, those constructs containing the chromo domains consistently displayed higher rates of ATPase activity than did the ATPase-helicase (AH) domain alone (Fig. 2). These observations argue for a role for the chromo domains in stimulating the ATPase (and potentially nucleosome remodeling) activity, perhaps by directly binding DNA and thereby facilitating the movement of CHD4 along DNA. Indeed, the chromo domains within the *Drosophila melanogaster* CHD4 homolog Mi-2 have been shown to be capable of binding to DNA, and it is possible that the chromo domains of human CHD4 also retain this feature.^16^ The enzymatic parameters *K*_m_ and *V*_max_ for the ATPase activity of the longest CHD4 derivative, PP-CC-AH-D, were found to be 1.34 mM and 16.9 pmol/min, respectively (Supplementary Table 1). This experimentally determined *K*_m_ value is ~ 10-fold greater than that reported for the native human NuRD complex, suggesting that other NuRD subunits may be required for CHD4 to function most efficiently as an ATPase in the presence of nucleosomes.^11^

Previous peptide binding studies have suggested that the N-terminal tail of histone H3 binds to the PHD domains in CHD4 and that methylation of lysine 4 inhibits this activity.^6,7^ More recently, it has been proposed that the two PHD domains simultaneously recognize the two histone H3 tails in the same nucleosome to mediate the repressive functions of CHD4.^17^ We therefore compared the ATPase activity of CHD4 on unmodified and histone H3K4me1- and histone H3K4me3-modified nucleosomal substrates. In the presence of unmodified nucleosomes, but not in the presence of methylated nucleosomes, an increase in ATPase activity is observed for the construct having the PHD domains, suggesting that the interaction of the PHD domains with unmodified histone H3 further enhances the activity of the ATPase (Fig. 2b). Consistent with previous peptide binding experiments, however, H3K4 methylation seems to prevent this.^6,7^

To investigate whether the PHD domains similarly influence the chromatin remodeling function of CHD4, we performed nucleosome-sliding assays with our PP-CC-AH-D and CC-AH-D recombinant proteins. The longest recombinant CHD4 protein PP-CC-AH-D is able to move nucleosomes along DNA, with a directionality of repositioning similar to that previously reported for dMi-2.^16^ Clearly, the PHD domains are important for this nucleosome repositioning, because the sliding activity of the construct lacking these domains is diminished relative to PP-CC-AH-D (Fig. 2c–e).

While the ATPase and remodeling activities of the PHD-containing CHD4 construct in the presence of nucleosomes appear to be enhanced compared to the corresponding CC-AH-D construct lacking these domains, the PHD-containing construct binds nucleosomes more weakly (Fig. 2). How can this be explained? The association of nucleosomes with CHD4 is likely to be mediated through the binding of the PHD domains to histone H3, together with interactions of the chromo and ATPase-helicase domains with DNA. Although the isolated PHD domains of CHD4 clearly bind histone H3, we reasoned that if either the PHD domains or the ATPase-helicase motif are inaccessible in intact CHD4, this might lead to a weak interaction with nucleosomes. In particular, we wondered whether it is possible that CHD4 exists in an inactive nucleosome binding conformation, which rearranges as CHD4 begins to function as an active ATPase/remodeler, permitting the association of the chromo domains and ATPase-helicase motif with DNA and nucleosomes, thereby enhancing its activity. Potentially, this could involve a conformational rearrangement of the PHD and chromo domains with respect to the ATPase-helicase motif, but other possibilities can also be envisaged.

### SAXS analyses of CHD4

The results of the ATPase assays described above suggested that the PHD and/or the chromo domains may interact with the ATPase domain of CHD4, thus modulating its activity. To test this hypothesis, we undertook small-angle X-ray scattering (SAXS) studies to identify domain interactions.

**Fig. 3:**
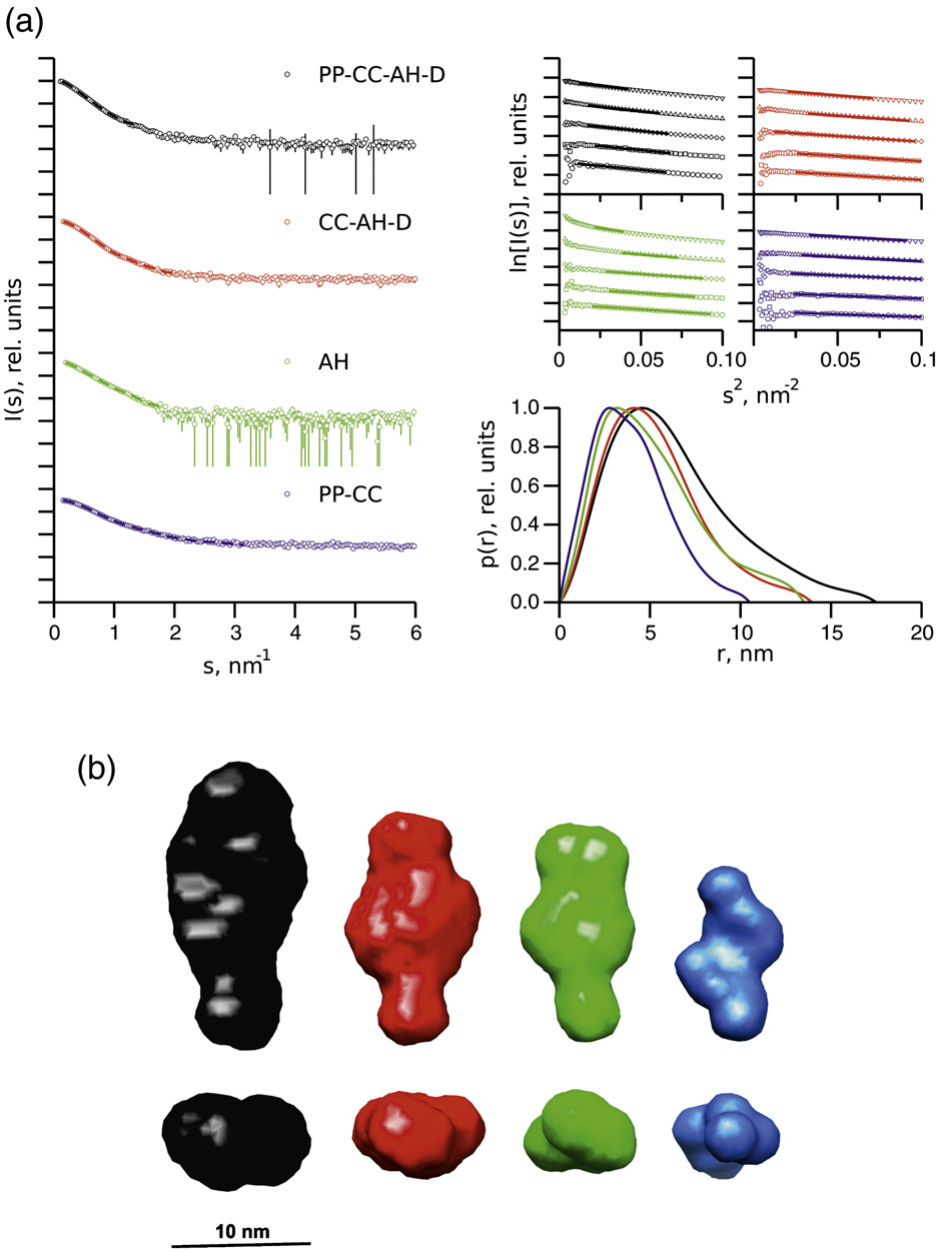

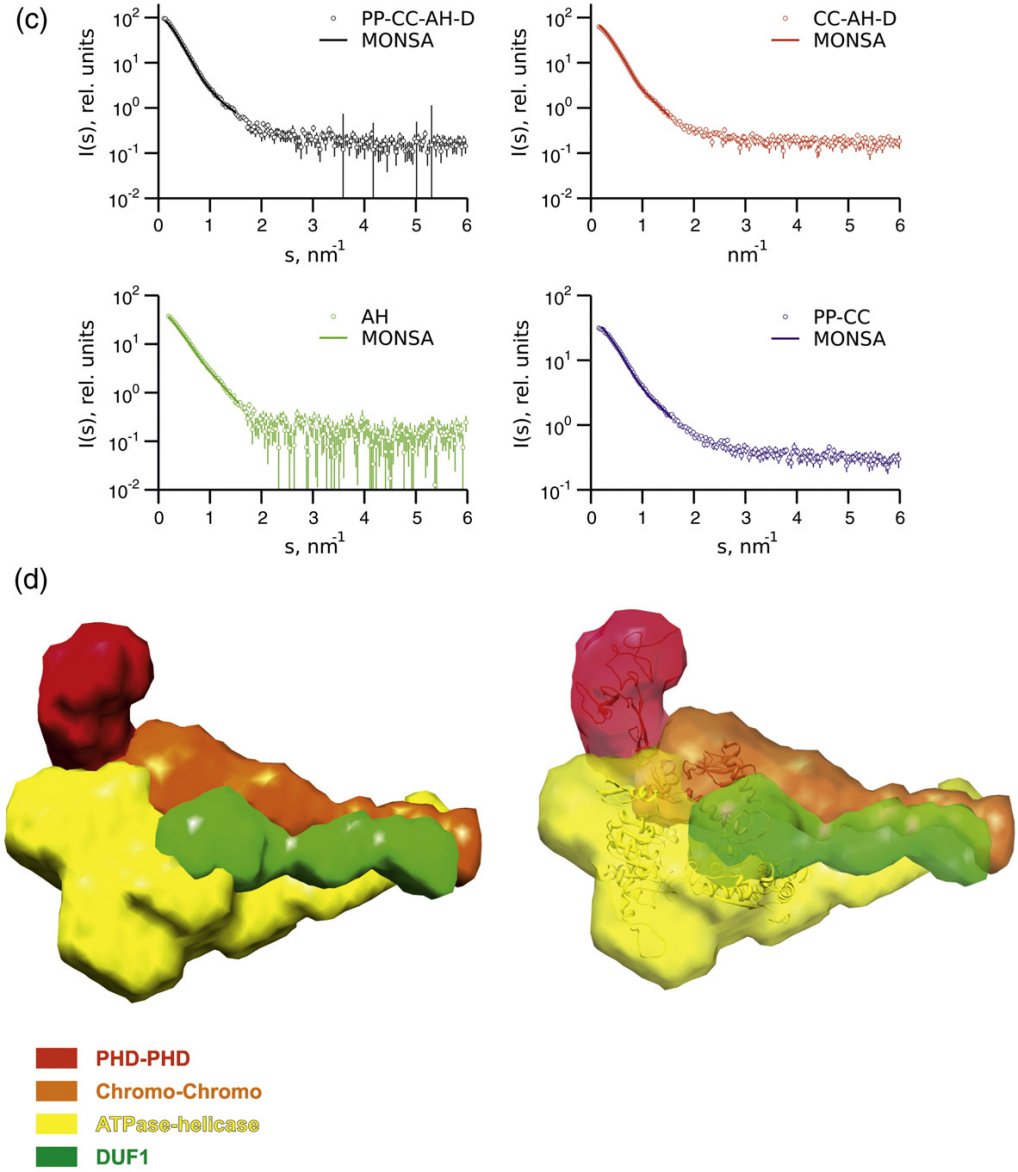

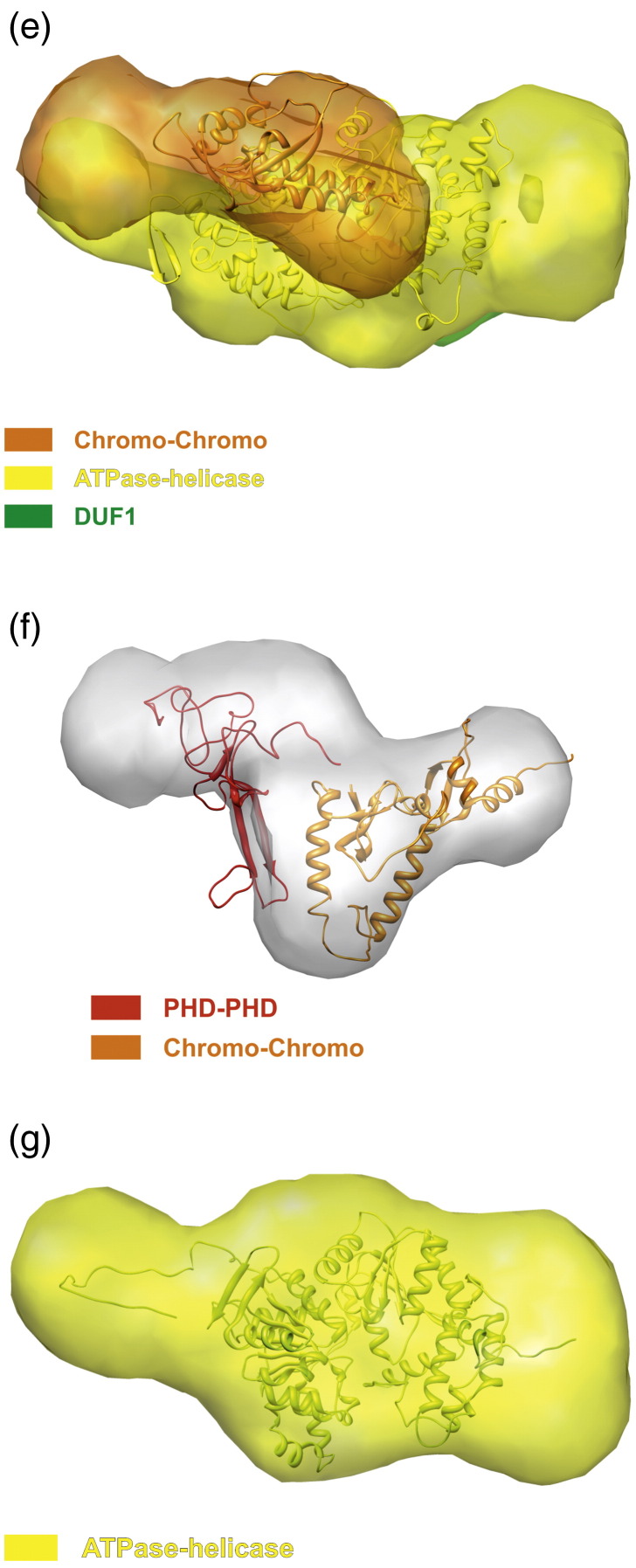
SAXS analyses of CHD4. (a) SAXS profiles (left), Guinier plots (top right), and distance distribution *p*(*r*) functions (bottom right) are shown for the CHD4 constructs PP-CC-AH-D (black), CC-AH-D (red), AH (green), and PP-CC (blue). Guinier fits for *R*_g_ and *I*(0) estimation are shown where the linear regions defining *s*_min_ and *s*_max_ are represented as thick lines. (b) The *ab initio* bead models for each construct are represented using the same color scheme as in (a) and are displayed as surfaces. A scale bar is indicated, and the representations in the bottom panel are related to those in the upper panel by a rotation of 90° counterclockwise around the *z*-axis. (c) The fits of the MONSA shapes shown in (b) to the processed experimental scattering data for each construct are shown as continuous lines with the same color scheme as in (a). (d) The MONSA shapes for the model of the complete construct PP-CC-AH-D are shown with (right) and without (left) the homology model superimposed. (e) The MONSA shapes for the construct CC-AH-D are presented with the homology model superimposed. (f) The *ab initio* beads model for the PP-CC construct (white) is shown with the CORAL rigid-body model superimposed. (g) The *ab initio* bead model for the AH construct is displayed with the ATPase homology model (yellow) superimposed. These homology models were generated using the SWISS-MODEL Comparative Protein Modeling program based on the structures of CHD4 and CHD1 (Protein Data Bank IDs 1MM2, 2L5U, 2EE1, and 3MWY). For all models, PHD (P) domains are colored red, chromo (C) domains are colored orange, the ATPase-helicase (AH) domain is colored yellow, and DUF1 (D) is colored green.

The overall shape and size parameters extracted from the SAXS data are summarized in Table 1, Fig. 3a, and Supplementary Fig. 2. The estimated molecular mass (MM) and radius of gyration (*R*_g_) extracted from Guinier plots (Fig. 3a) slightly increase with concentration, indicating that a concentration-dependent self-association is observed for each construct. However, the MM for the most dilute samples is close to that expected for a monomeric species, and the data were subsequently merged and extrapolated to infinite dilution to remove the contribution due to interparticle interactions from the analysis.^18^ The distance functions *p*(*r*) describing the distribution of interatomic scattering vectors within each construct are in each case skewed towards large distances, typical for extended structures. Interestingly, the maximum dimensions *D*_max_ of both the CC-AH-D and AH constructs are similar (~ 14.5 nm), suggesting that the addition of the chromo and DUF1 domains does not increase the length of the CC-AH-D construct relative to the ATPase alone, raising the possibility that these domains fold back towards (and possibly form contacts with) the ATPase domain.

Shape envelopes were reconstructed from the SAXS data for constructs with and without the N-terminal PHD and chromo domains using both a single- and multiphase modeling approach that has been successfully applied to similar multidomain modular systems.^19,20^ *Ab initio* single-phase shape reconstructions using the bead modeling program DAMMIF reveal extended structures for each construct (Fig. 3a and b and Supplementary Fig. 1), as suggested by the *p*(*r*) functions.^21^ These models indicate that there must be some contacts formed between domains in order for the CC-AH-D, AH, and PP-CC shape envelopes to fit into the PP-CC-AH-D shape envelope in any meaningful way. In order to explore this further, we used the multiphase bead modeling program MONSA to generate a model of the intact structure of CHD4 encompassing all domains from the start of the first PHD domain to the end of the first domain of unknown function, DUF1 (Fig. 3c and d).^21^ The simultaneous fits of the resulting model to the scattering data are good (discrepancies: 1.0 < χ < 1.9), suggesting that there exists no significant conformational change of the subconstructs relative to PP-CC-AH-D (at the resolution of SAXS studies). Composite homology models of the PP-CC-AH-D and CC-AH-D constructs were assembled and fitted into the corresponding shape envelopes (Fig. 3d and e). In the composite PP-CC-AH-D (Fig. 3d) and CC-AH-D (Fig. 3e) models, the ATPase domain is seen to associate with the tandem chromo domains (Fig. 3d). Additionally, contacts are observed between the PHD and chromo domains, between the ATPase domain and the DUF1, and between the chromo domains and the DUF1 (Fig. 3d).

To examine the conformation of the PHD and chromo domains in the absence of the ATPase domain itself, and using the available high-resolution structures of these individual domains, we performed rigid-body modeling using the SAXS data of the PP-CC construct using the program CORAL (COmplexes with RAndom Loops) (Fig. 3f).^22^ The model generated fits the data (χ = 1.1) and overlays well with the zigzag *ab initio* shape envelope generated independently (Fig. 3f). In this model, the tandem PHD and chromo domains adopt the same orientation relative to each other as they do in the context of the larger construct. The results suggest that the PHD and chromo domains in CHD4 function as a unit, and it is possible that the interaction between the tandem PHD and chromo domains promotes association of the latter with the ATPase domain, for example, by stabilizing the structure of the chromo domains, and that this may play a role in regulating the function of CHD4. A comparison of the composite PP-CC-AH-D and CC-AH-D models (Fig. 3d and e) to the models of the ATPase alone (Fig. 3g) suggests that the chromo and DUF1 domains fold onto the core of the ATPase.

### The PHD and chromo domains of CHD4 associate with its ATPase motif

**Fig. 4:**
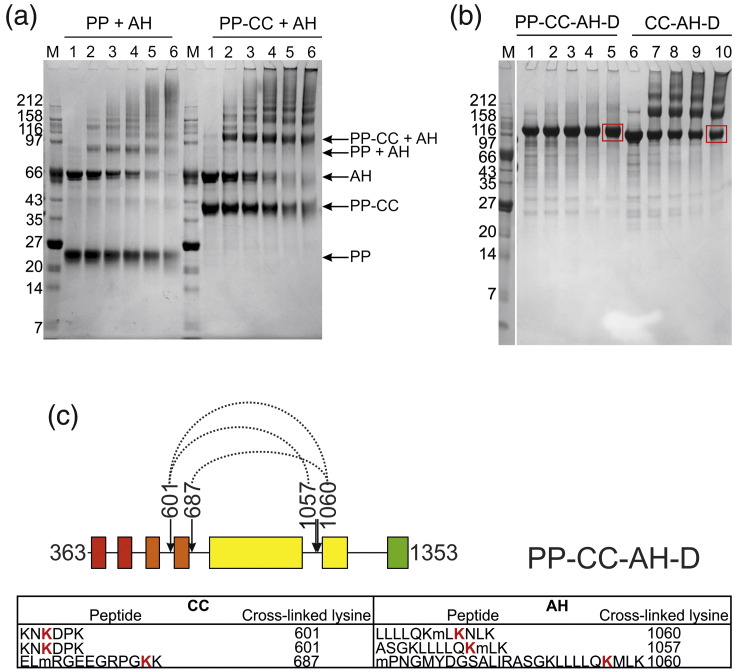
Interdomain cross-links in CHD4. (a) The ATPase domain of CHD4 (AH) was mixed with either the tandem PHD domains (PP) or the tandem PHD plus tandem chromo domains (PP-CC) at a 1:1 molar ratio, and was then incubated with H_12_/D_12_-labeled BS^3^ cross-linker in the following protein:cross-linker molar ratios: 1:0 (lane 1), 1:20 (lane 2), 1:50 (lane 3), 1:100 (lane 4), 1:200 (lane 5), and 1:500 (lane 6) for 30 min at room temperature. The protein molecular mass marker (M) is NEB Broad Range. Each reaction was stopped by the addition of 1/10 volume of 1 M Tris, pH 8.0, for 15 min. The reaction mix was separated on a NuPAGE 4–12% Bistris gel and stained with Coomassie Brilliant Blue stain. (b) The above procedure was repeated for constructs PP-CC-AH-D and CC-AH-D separately, each at the following protein:cross-linker ratios: 1:0 (lanes 1 and 6), 1:20 (lanes 2 and 7), 1:50 (lanes 3 and 8), 1:100 (lanes 4 and 9), and 1:200 (lanes 5 and 10). SDS-PAGE bands from lanes 5 and 10 found to have a molecular mass consistent with that of either monomeric PP-CC-AH-D or monomeric CC-AH-D, respectively, are indicated in red boxes. (c) The indicated gel bands were excised, trypsinized, and subjected to LC–MS/MS analysis. Cross-links between the chromo and ATPase-helicase domains within each construct were identified using Xlink-Identifier and are indicated by dotted lines. The sequence of each cross-linked peptide is tabulated below, where each modified lysine is represented in bold, red typeface. The residue numbers of these modified sites are indicated within the table and above the figure. Oxidized methionine residues are denoted in lower case.

To further examine whether the PHD and chromo domains interact directly with the ATPase domain of CHD4, a 1:1 protonated/deuterated (h_12_/d_12_) mixture of the cross-linker BS^3^ was added to two separate samples that contained the ATPase domain construct (AH) in complex with either the tandem PHD domain construct (PP) or the construct including the tandem PHD and tandem chromo domains (PP-CC). In both cases, the PHD-containing construct (either PP, or PP-CC) formed cross-linked complexes with the ATPase-helicase construct (AH) (Fig. 4a). However, the sample containing both the PHD and chromo domains displayed a significantly greater level of cross-linking (as assessed by the gel band intensity of the cross-linked species) than did the sample containing the PHD domains alone, suggesting that the chromo domains interact with the ATPase domain. The double chromo domains of the related CHD1 remodeler have recently been shown to prevent DNA binding and thereby inhibit the ATPase motor.^23^ They have also been shown to mediate ATP-dependent nucleosome mobilization in the *D. melanogaster* homolog of CHD4, dMi-2, and it is possible that the tandem PHD and chromo domains of human CHD4 may also play a regulatory role.^16^

We then used cross-linking/mass spectrometry to identify interdomain contacts within the separate intact protein constructs PP-CC-AH-D and CC-AH-D (Fig. 4b). After incubation with the cross-linker, SDS-PAGE bands corresponding to the monomeric molecular masses of each of these constructs were excised, trypsinized, and analyzed using liquid chromatography–tandem mass spectrometry (LC–MS/MS), and the cross-linked peptides were identified using the Xlink-Identifier data analysis platform.^24^ Interestingly, the presence of the double PHD domains appears to inhibit the self-association of CHD4 at higher molar ratios of cross-linker to protein (Fig. 4b). The BS^3^ cross-linker covalently connects primary amine groups (on lysine residues or on the N-terminus of a particular polypeptide) within a distance of 12 Å. For both protein constructs, data sets were collected where ions with charge states of either 2+ and above or 4+ and above were selected for fragmentation. Across all four data sets, we observed mostly the same cross-links for residues contained within the chromo domains, ATPase-helicase domain, and the DUF1. From the two PP-CC-AH-D data sets, several cross-links were obtained between peptides from the PHD domains and peptides from the chromo domains (data not shown), suggesting that these domains do associate. We also identified several cross-links between peptides from the chromo domains and the ATPase-helicase (Fig. 4c). This is in agreement both with our SAXS model of CHD4 and with the crystal structure of CHD1. The presence of several cross-links between the DUF1 and both the ATPase and the chromo domains further validates our SAXS model.

These findings suggest that the chromo domains associate with the ATPase-helicase portion of CHD4 and that the DUF1 interacts with both of these motifs.

### Limited proteolysis and mass spectrometric analysis of CHD4

**Fig. 5:**
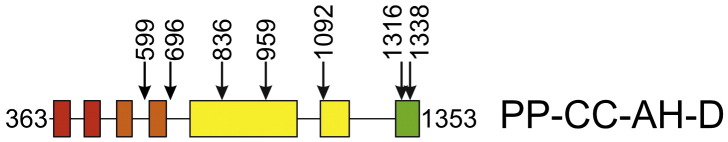
Limited proteolysis of the PHD, chromo, ATPase-helicase, and DUF1 domains of CHD4. Sites of proteolytic cleavage following LC–MS/MS on construct PP-CC-AH-D are indicated by black arrows with the residue numbers shown above. PHD (P) domains are colored red, chromo (C) domains are colored orange, the ATPase-helicase (AH) domain is colored yellow, and the domains of unknown function (D) are colored green.

The longest of the CHD4 constructs described in this study (PP-CC-AH-D) was subjected to limited proteolysis followed by LC–MS/MS so as to identify potentially stable fragments and unstructured loop regions (Fig. 5). The first tryptic cleavage occurred immediately following the addition of trypsin and split the protein into two segments at a potentially flexible region in the middle of the ATPase domain (at residue 836) (Fig. 5). The DUF1 domain in the C-terminal half of the cleaved protein was then progressively degraded over the time course (from residue 1353 to 1338 and then back to residue 1316). This suggests that the DUF1 segment may be unstructured, or become unstructured following cleavage of the ATPase, consistent with our SAXS models, which imply that the DUF1 folds over to associate with the ATPase domain. This C-terminal segment of CHD4 was then further cleaved at sites within the helicase domain (at residue 1092 and then 959). The N-terminal half of the bisected protein appeared to be less susceptible to tryptic digestion and only underwent further degradation to residue 696 (after the second chromo domain) and at the linker region between the two chromo domains (residue 599) (Fig. 5). These results suggest that the ATPase motor of CHD4 contains a flexible linker between its two lobes, in agreement with the structure of the CHD1 ATPase. Additionally, the N-terminal portion of CHD4 containing the tandem PHD and chromo domains is comparatively resistant to proteolytic digestion, suggesting that these domains associate to produce a stable structure with no significant flexibility between the domains, consistent with our cross-linking/MS results and the SAXS models for PP-CC and PP-CC-AH-D.

These structural data support the conclusions from the ATPase/remodeling assays and additionally imply an interaction between the PHD and chromo domains. The PHD domains of CHD4 have previously been shown to individually recognize the unmodified N-terminal tail of histone H3, and together they have been suggested to recognize the two histone H3 tails in a single nucleosome.^6,7,17^ Our results are consistent with a model whereby the PHD domains make intramolecular interactions with the chromo domains, which together limit the binding of CHD4 to nucleosomes (Fig. 2a), but that when they do bind histone H3, this may stimulate the activity of the enzyme (Fig. 2b). This activation mechanism will likely involve a structural rearrangement of the protein, whereby the PHD and chromo domains release the ATPase-helicase domain to allow access to DNA. Such a conformational change may represent a common control mechanism for other PHD domain-containing CHD family chromatin remodelers, which, in the case of CHD4, might be influenced by other subunits of the NuRD complex to modulate transcription.

## Materials and Methods

### Cloning, expression, and purification

Full-length recombinant human CHD4 proved to be a suboptimal candidate for expression and purification from insect cells. Therefore, constructs spanning the following residues of isoform 2 of human CHD4 (Fig. 1a) were cloned into the baculovirus transfer vectors pFB-CT10HF-LIC or pFB-LIC-Bse, which contain either an N-terminal His_6_ or a C-terminal His_10_ tag alongside an appropriately located TEV protease cleavage site: 363–1353 (PP-CC-AH-D), 494–1353 (CC-AH-D), 685–1233 (AH), 363–512 (PP), 363–682 (PP-CC), and 363–1226 (PP-CC-AH).^25^ Recombinant bacmid DNA was generated from each of these transfer plasmids. Proteins were expressed in Sf-9 cells at a volume of 1.5 L each following infection with P-III baculoviral stocks made from these bacmids.^26^ Sf-9 cells were seeded into 1.5 L of Insect-Xpress medium at a density of 3 × 10^6^ cells/mL. Cells were cultured at 27 °C with agitation at 120 rpm and were harvested 48 h postinfection by centrifugation at 900*g* for 25 min. Cell pellets were washed once in phosphate-buffered saline and stored at − 80 °C. On the day of purification, these cell pellets were thawed rapidly at 37 °C, resuspended in a buffer consisting of 50 mM Hepes, pH 7.5, 300 mM NaCl, 5% (v/v) glycerol, and 1 mM tris(2-carboxyethyl)phosphine supplemented with protease inhibitor cocktail III (Merck) and benzonase nuclease (Merck), and lysed by sonication on ice. Cell debris was pelleted by centrifugation at 21,000 rpm for 1 h at 4 °C, and the supernatant was subsequently filtered and incubated with nickel-IDA resin for 60 min at 4 °C with agitation at 3 rpm. Following several wash steps, each desired protein construct was eluted from the resin using the lysis buffer described above supplemented with 50–300 mM imidazole. Where appropriate, the hexahistidine tag was removed from each protein construct by overnight treatment with TEV protease at 4 °C. The cleaved CHD4 protein was concentrated to a volume of 5 mL and loaded onto a HiLoad Superdex 200 16/60 Prep Grade filtration column (GE Healthcare) equilibrated in 50 mM Hepes, pH 7.5, 300 mM NaCl, and 5% (v/v) glycerol, with 1-mL fractions collected at a flow rate of 1 mL/min at 4 °C.

The purity and identity of each protein construct were confirmed by SDS-PAGE and electrospray ionization time-of-flight mass spectrometry (Agilent LC/MSD). Protein monodispersity was determined by dynamic light scattering.

Recombinant modified histones and biotinylated nucleosomes were prepared and assembled as described elsewhere.^27^

### Nucleosome binding dot blots

Two hundred fifty nanograms of each of the following CHD4 protein constructs was spotted onto two nitrocellulose membranes: PP-CC-AH-D, CC-AH-D and AH. Two hundred fifty nanograms of biotinylated unmodified nucleosomes was also spotted onto each membrane to serve as a positive control for streptavidin binding, alongside 250 ng of an unrelated negative control protein. The membranes were blocked with 5% bovine serum albumin (BSA) in a solution of 20 mM Tris–HCl, pH 7.5, 150 mM NaCl, and 0.05% Tween-20 (TBS-T), washed three times in TBS-T, and then incubated with 1 μg of biotinylated unmodified nucleosomes in 0.1% BSA in TBS-T for 1 h at room temperature with agitation. After washing three times with TBS-T, streptavidin-HRP was added to the membrane for an additional 60 min at room temperature with gentle agitation. After washing, the membranes were developed with an enhancement chemiluminescence substrate. The background-subtracted pixel intensities of each spot were plotted as a histogram.

### ATPase assays

Standard ATPase assay reaction mixtures (50 μL) contained 10 mM Tris–HCl, pH 7.5, 1 mM DTT, 6 mM MgCl_2_, 5% glycerol, a trace amount of [γ-^32^P]ATP (∼ 2 nM) mixed with 0.5 mM cold ATP, 7 μM of each CHD4 construct, and where indicated, 16 μM DNA species or 3 μM recombinant nucleosomes. The double-stranded nucleosomal DNA used was a biotinylated 185-bp fragment containing the 601 nucleosome positioning sequence.^27,28^ The reactions were initiated by the addition of the ATP mixture and incubated at 37 °C for 30 min. Samples (1 μL) were removed and evaluated by thin-layer chromatography as described previously.^29^ Less than 35% of the ATP substrate was consumed in the reaction over the entire time course of the experiment. The results of three experiments are presented.

For the measurement and calculation of enzymatic parameters for the ATPase activity of CHD4, 12 standard 50-μL ATPase assay reaction mixtures were prepared as above and were supplemented with 0.43 μM PP-CC-AH-D CHD4 protein and 32.6 nM recombinant nucleosomes at a range of concentrations of cold ATP (ranging from 0.007 to 2 μM final concentration). Reactions were initiated by the addition of the ATP mixture and incubated at 37 °C for 20 min. Samples (0.5 μL) were removed and evaluated by thin-layer chromatography as above. Each reaction was repeated in triplicate. Programs from the GraphPad software package were used for the determination of the enzymatic parameters *K*_m_ and *V*_max_ by nonlinear least-squares regression (GraphPad Prism; GraphPad Software Inc.) using the Michaelis–Menten equation: EP (pmol/mg)/EP_max_ [ATP]/(*K*_m_^h^ + [ATP]) in which the maximum amount of EP (enzyme–product complex) formed is EP_max_, and *K*_m_^h^ is the concentration of ATP required for the half-maximal formation of EP.

### Nucleosome sliding electrophoretic mobility shift assays

Nucleosomes were assembled on a Cy3-labeled 0W47 DNA fragment containing the strong Widom sequence for nucleosome positioning (147 bp) and 47 bp DNA on one side extending the nucleosome. CHD proteins (0.3 μM) were incubated with 0.1 μM nucleosomes in the presence (+) or absence (−) of 1 mM ATP in 30 mM NaCl, 3 mM MgCl_2_, and 50 mM Tris, pH 8.0, for 30 min at 37 °C. NaCl (160 mM), 2.5% sucrose, and 1 μg plasmid DNA were added before each assay was loaded onto a 5% 0.2× TBE [Tris–borate–EDTA (ethylenediaminetetraacetic acid)] native polyacrylamide gel run in 0.2× TBE for 3.5 h at 300 V. The gels were scanned for the Cy3 signal using a phosphoimager. These assays were repeated using a dilution series ranging from 0.05 to 0.2 μM CHD4 protein and 0.013 μM nucleosomes (0w47) in 40 mM NaCl, 10 mM MgCl_2_, and 50 mM Tris, pH 8.0, in the presence (+) or absence (−) of 1 mM ATP for 1 h at 37 °C. NaCl (160 mM), 2.5% sucrose, and 4 μg plasmid DNA were added prior to loading each reaction onto the gel. The automatic imaging data analysis program AIDA (Raytest, Straubenhardt, Germany) was used to quantify the band intensity, where 100% is expressed as the sum of the two nucleosomal bands in each reaction. Each histogram represents the upper (i.e., repositioned) nucleosomal band in each reaction as a percentage of this sum. Yeast CHD1 was purified from a CHD1-TAP strain (Euroscarf) as previously reported.^30^

### SAXS data collection and model generation

The following constructs of CHD4 were used for SAXS studies: PP-CC (spanning the tandem PHD and tandem chromo domains), AH (the ATPase-helicase construct), PP-CC-AH-D (encompassing all domains from the first PHD domain to the first domain of unknown function, DUF1), and CC-AH-D (from the first chromo domain to the end of DUF1). SAXS data were collected at the X33 beamline at the European Molecular Biology Laboratory/Deutsches Elektronen-Synchrotron (Hamburg) at a temperature of 11 °C using a camera length of 2.7 m covering a range of momentum transfer 0.08 ≤ *s* ≤ 6.0 nm^− 1^ (*s* = 4π sinθ/λ, where 2θ is the scattering angle, and λ = 0.15 nm is the X-ray wavelength) and were subsequently processed using PRIMUS.^21,31,32^ Distance distributions, *p*(*r*), providing a real-space interpretation of the SAXS data were determined using the indirect Fourier transform method implemented in the program GNOM.^33^ The samples were prepared in concentration ranges from 1.04 to 8.56 mg/mL in 50 mM Hepes, pH 7.5, 300 mM NaCl, and 5% (v/v) glycerol. Solute masses were calculated by comparing the intensity of the forward scattering with that of a solution of BSA as a reference. Low-resolution shape models for CHD4 were reconstructed using the program DAMMIF and the multiphase equivalent program MONSA.^21^ Here, each particle is represented as an array of densely packed beads within a sphere whose diameter is equal to *D*_max_. In DAMMIF, each of these beads is assigned to either the particle (as a simple “phase”) or to the solvent. In MONSA, each bead is assigned either to the solvent (index = 0) or to one of the parts of the complex (index = 1 corresponding to PP, index = 2 to CC, index = 3 to AH, and index = 4 to D). The particle is, therefore, represented at low resolution by four phases (portions of the protein), and the overall model can be defined as a string of length *M* containing the phase index for each bead. Simulated annealing calculations using both DAMMIF and MONSA were carried out to search for a model composed of interconnected compact phases, fitting single (DAMMIF), or multiple (MONSA) curves to minimize the overall discrepancy (χ):^21^

$$x^{2}=\sum_{k}\frac{1}{N-1}\sum_{j}\left[\frac{I_{\exp}\left(s_{j}\right)-cI_{\text{calc}}\left(s_{j}\right)}{\sigma \left(s_{j}\right)}\right]$$

where *N* is the number of experimental points; *I*_exp_(*s*_*j*_) and *I*_calc_(*s*_*j*_) are the experimental and calculated scattering intensities, respectively; *c* is a scaling factor; and σ(*s*_*j*_) is the experimental error at the momentum transfer *s*_*j*_.

Multiple DAMMIF models from 10 independent reconstructions for each CHD4 construct were clustered using the program DAMCLUST (Petoukhov *et al.*, submitted) according to a similarity measure, the normalized spatial discrepancy.^34^ An average model for each cluster was generated and filtered for regions of high volume occupancy.^35^

Rigid-body models of construct PP-CC were generated from the high-resolution structures of the chromo and PHD domains (Protein Data Bank IDs 1MM2, 2L5U, and 2EE1) using the program CORAL.^22^ Composite homology models of the different CHD4 constructs were constructed using the SWISS-MODEL Comparative Protein Modeling program.^36^

### Cross-linking with BS^3^

The CHD4 protein construct AH was mixed with either construct PP or PP-CC at a 1:1 molar ratio to give a final concentration of 5–10 μM in 50 mM Hepes, pH 7.5, 300 mM NaCl, and 5% (v/v) glycerol. H_12_/D_12_-isotope-labeled BS^3^ cross-linker (Creative Molecules Inc.) was added at the following protein:cross-linker molar ratios: 1:0, 1:20, 1:50, 1:100, 1:200, and 1:500 for 30 min at room temperature. Each reaction was stopped by the addition of 1/10 volume of 1 M Tris, pH 8.0, for 15 min at room temperature. Cross-linked species were resolved on a NuPAGE 4–12% 2-[bis(2-hydroxyethyl)amino]-2-(hydroxymethyl)propane-1,3-diol (Bistris) gel that was then stained with Coomassie Brilliant Blue. The same procedure was employed to analyze interdomain cross-links within a single CHD4 protein construct (either construct PP-CC-AH-D or CC-AH-D).

### Identification of cross-linked peptides by MS/MS

LC–MS/MS experiments were performed using an Eksigent NanoLC-1D Plus (Eksigent Technologies, Dublin, CA) HPLC system connected to an LTQ Orbitrap Velos mass spectrometer (ThermoFisher, Waltham, MA). Peptides were loaded via an autosampler onto a pre-column (Dionex Acclaim PepMap 100 C18, 5 μM particle size, 100 Å, 300 μM i.d. × 5 mm) in 0.1% formic acid at 10 μL/min and were then eluted onto an analytical LC Packings (Dionex, Sunnyvale, CA) PepMap 100 column (C18, 75 μM i.d. × 150 mm, 3 μM particle size) for resolution using reverse-phase chromatography 5 min later. Here, a gradient of 5–50% of a solution of 0.1% formic acid in acetonitrile was applied over a 45 min period. This eluent was subsequently sprayed into the mass spectrometer via a New Objective nanospray source, and *m*/*z* values were measured with an Orbitrap Velos mass analyzer, set at a resolution of 30,000. Fragment ions were generated by collision-induced dissociation in the linear ion trap, and ions with charge states of either 2+ and above or 4+ and above were selected for fragmentation. These data were processed using Protein Discoverer (version 1.2., ThermoFisher) and were converted to mgf, dta, and cdf files for analysis. The Xlink-Identifier data analysis platform was used to identify intrapeptide, interpeptide, and dead-end cross-links.^24^ The presence of the PP, PP-CC, and AH species in the corresponding marked bands was verified by MASCOT mass spectrometry peptide coverage analysis.

### Limited proteolysis and mass spectrometry

The PP-CC-AH-D construct of CHD4 was incubated for 180 min at 22 °C with trypsin at a 125:1 CHD4 protein:trypsin molar ratio. Sample aliquots were taken for SDS-PAGE and LC–MS/MS at 0, 10, 20, 30, 60, 120, and 180 min following the addition of trypsin. Samples for SDS-PAGE were boiled immediately in standard Laemmli loading buffer and then separated by SDS-PAGE on a NuPAGE Novex Bistris 4–12% gel. Gel bands corresponding to stable domains were excised from the gel and overlaid with 10% MeOH at 4 °C. In-gel tryptic digests of each gel slice were performed as described previously.^37^ A Dionex U3000 nano-HPLC coupled to a Bruker Esquire HCT ion trap mass spectrometer was used to perform LC–MS/MS.^37^ Samples were also removed from the original tryptic digest reaction for intact mass analysis at the same time points. These samples were supplemented with 10% formic acid, and the intact mass of proteins in each sample was determined using an MSD-ToF electrospray ionization orthogonal time-of-flight mass spectrometer (Agilent).^37^

## Figures and Tables

**Table 1:** CHD4 SAXS parameters

Construct	Concentration (mg/mL)	*R*_g_^autoRg^ (nm)	*R*_g_^GNOM^ (nm)	*D*_max_ (nm)	MM_SAXS_ (kDa)
PP-CC-AH-D (117 kDa)	8.3	5.6 ± 0.1	5.7 ± 0.1	19.6 ± 0.5	142 ± 15
	4.9	5.3 ± 0.1	5.4 ± 0.1	18.4 ± 0.5	128 ± 10
	2.0	5.0 ± 0.1	5.2 ± 0.1	17.3 ± 0.5	121 ± 10
	1.0	5.0 ± 0.1	5.0 ± 0.1	17.1 ± 0.5	121 ± 10
	mer	4.9 ± 0.1	5.0 ± 0.1	17.4 ± 0.5	119 ± 10
CC-AH-D (101 kDa)	7.1	4.5 ± 0.1	4.6 ± 0.1	15.9 ± 0.5	93 ± 10
	4.1	4.3 ± 0.1	4.4 ± 0.1	14.9 ± 0.5	87 ± 10
	2.2	4.2 ± 0.1	4.2 ± 0.1	14.5 ± 0.5	81 ± 10
	1.0	4.0 ± 0.1	4.1 ± 0.1	14.2 ± 0.5	78 ± 10
	mer	4.0 ± 0.1	4.0 ± 0.1	14.5 ± 0.5	77 ± 10
AH (62.9 kDa)	8.6	6.5 ± 0.1	6.6 ± 0.1	21.9 ± 0.5	99 ± 10
	3.8	4.8 ± 0.1	5.0 ± 0.1	16.9 ± 0.5	66 ± 10
	2.1	4.4 ± 0.1	4.3 ± 0.1	14.7 ± 0.5	57 ± 10
	1.4	4.3 ± 0.1	4.2 ± 0.1	14.8 ± 0.5	53 ± 10
	mer	4.2 ± 0.1	4.0 ± 0.1	14.5 ± 0.5	52 ± 10
PP-CC (37.9 kDa)	8.2	4.0 ± 0.1	4.1 ± 0.1	13.9 ± 0.5	56 ± 10
	4.7	3.7 ± 0.1	3.9 ± 0.1	13.1 ± 0.5	49 ± 10
	2.7	3.5 ± 0.1	3.6 ± 0.1	12.2 ± 0.5	37 ± 10
	3.3	3.5 ± 0.1	3.6 ± 0.1	12.2 ± 0.5	46 ± 10
	1.1	3.2 ± 0.1	3.3 ± 0.1	11.3 ± 0.5	40 ± 10
	mer	3.0 ± 0.1	3.1 ± 0.1	10.5 ± 0.5	39 ± 10

*R*_g_^autoRg^ and *R*_g_^GNOM^ are the radii of gyration estimated from the SAXS data using the automated Guinier analysis routine and the GNOM program, respectively. *D*_max_ and MM_SAXS_ are the maximum particle dimension and molecular mass estimated from *I*(0). “mer” refers to the merged SAXS curve from all data sets that was extrapolated to infinite dilution.
