# Supplementary material for: The PHD and Chromo Domains Regulate the ATPase Activity of the Human Chromatin Remodeler CHD4
Source: J Mol Biol. 2012 Sep 7;422(1-2):3–17. doi: 10.1016/j.jmb.2012.04.031 (PMC3437443; doi:10.1016/j.jmb.2012.04.031)
Supplement: Supplementary file 1 — Supplementary data [file mmc1.docx]

**Supplementary Material.**

**A**

**
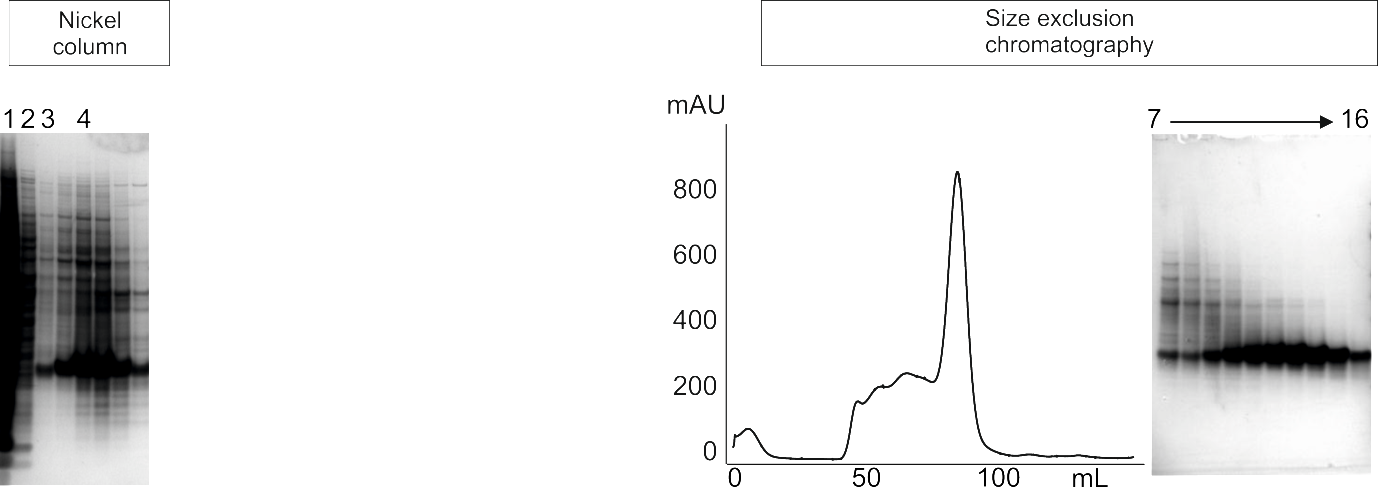
B
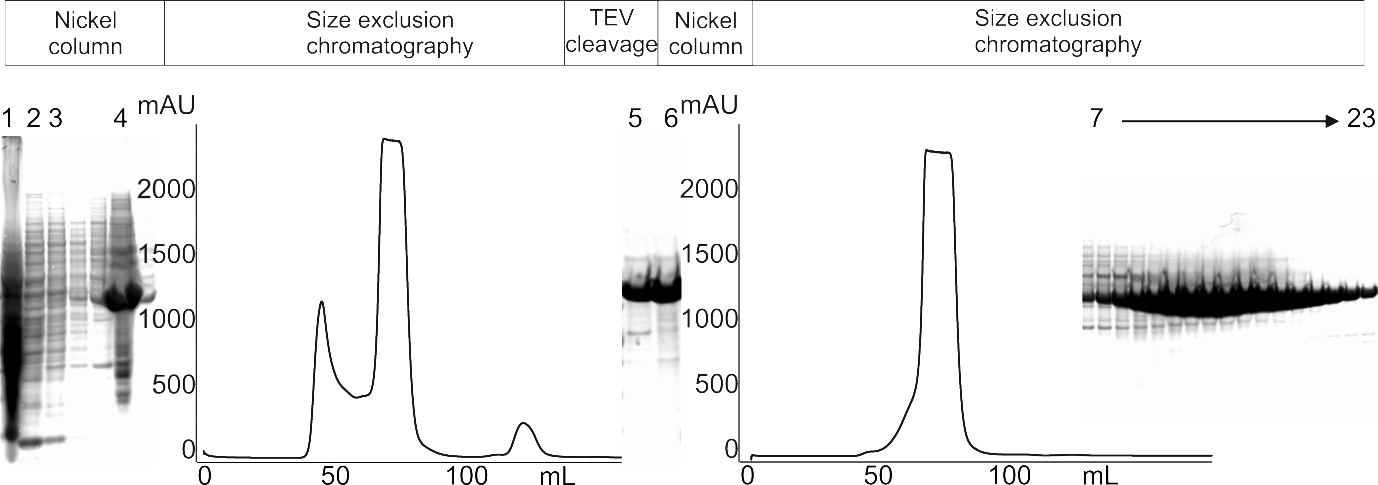
**

**C
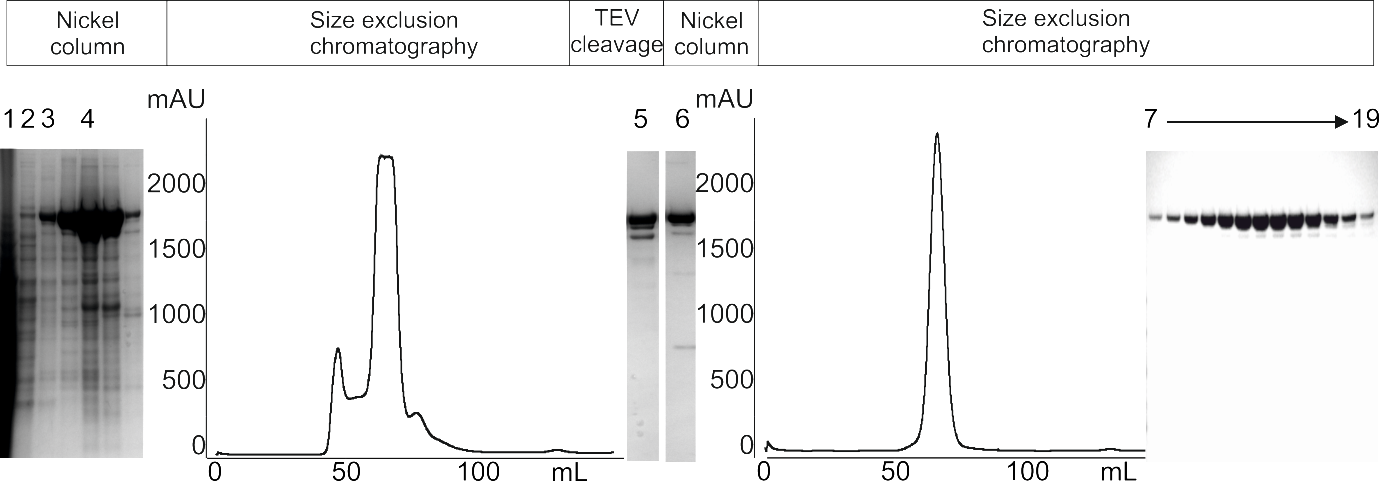
**

**D
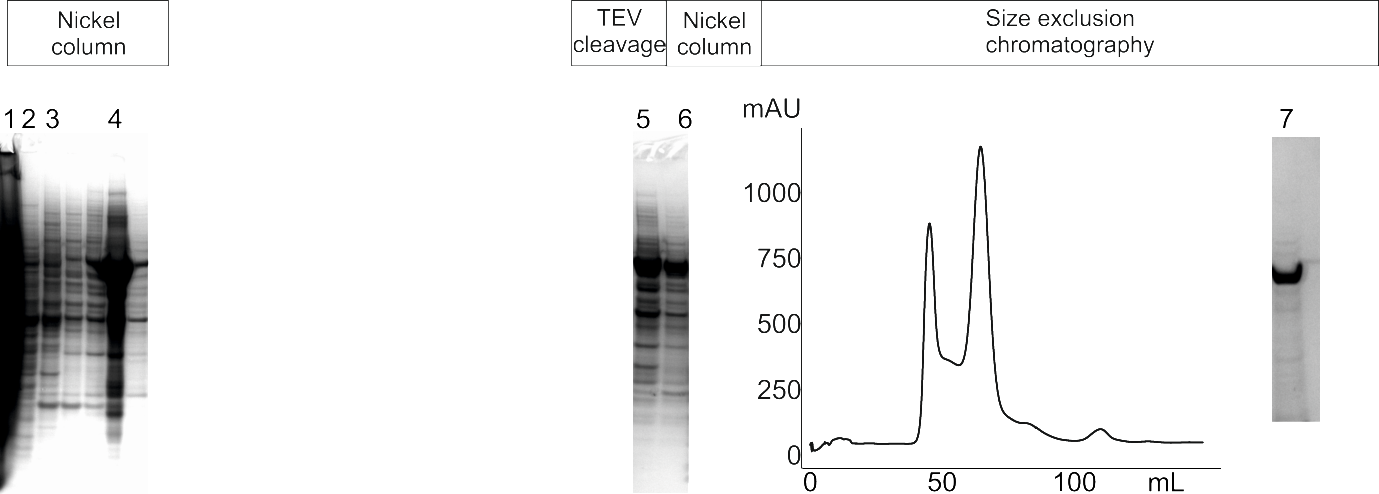
E**

**
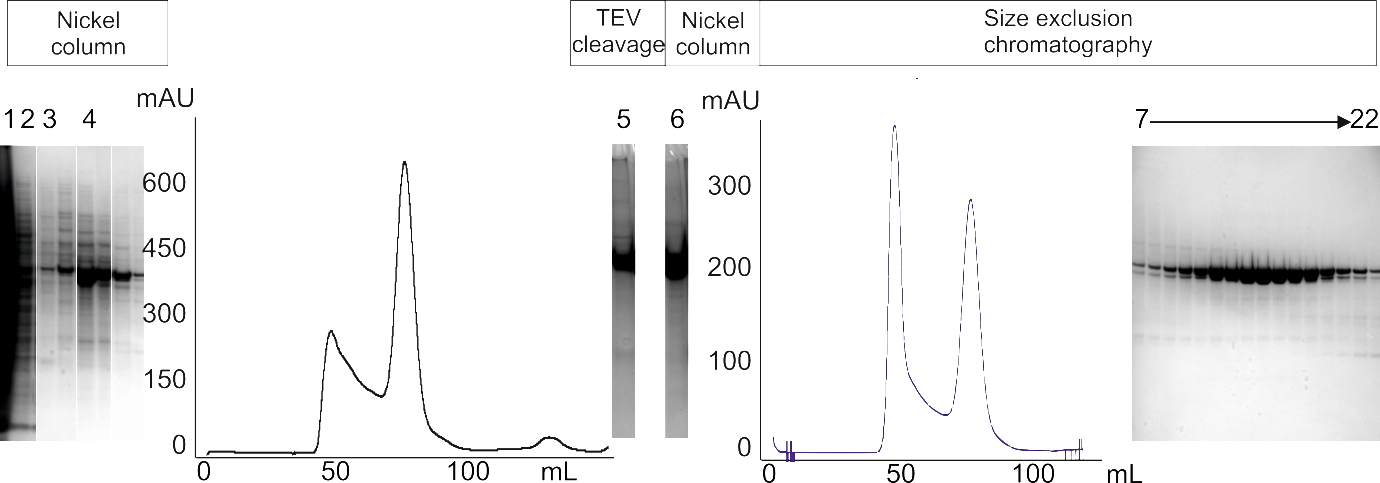
**

**Supplementary Figure 1. Purification strategy for CHD4 constructs PP (A), PP-CC (B), PP-CC-AH-D (C), PP-CC-AH (D), and AH (E).** Here, SDS PAGE gels are presented following the nickel affinity purification, TEV protease cleavage and size exclusion chromatography steps. Lane 1, total lysate; Lanes 2-3, the elutions from nickel beads using buffer supplemented with 10 and 30 mM imidazole, respectively; Lane 4, elutions from nickel beads using buffer supplemented with 100 (A, C, E) or 200 (B, D) mM imidazole, Lane 5, TEV-cleaved protein; Lane 6, flow-through following re-application of TEV-cleaved protein to a second nickel resin column; Lanes 7+, fractions from a peak with an elution volume corresponding to the predicted molecular mass of each CHD4 construct, eluted from a HiLoad Superdex 200 16/60 Prep Grade filtration column. Protein samples were resolved on a NuPAGE 4-12 % Bis-Tris gel stained with Coomassie Brilliant Blue stain.

**
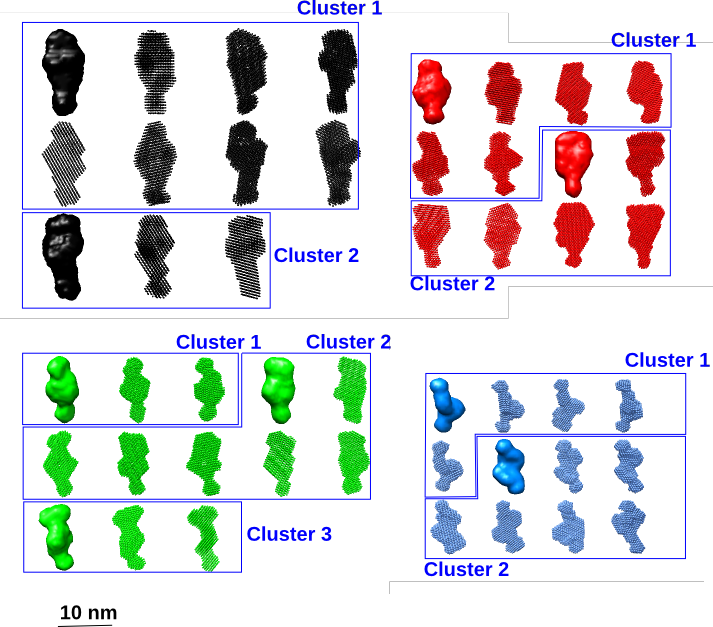
**

**Supplementary Figure 2.** **DAMMIF *Ab initio* modeling of CHD4.** The average filtered model for each cluster of shape reconstructions is shown as a surface and the individual cluster members shown as bead models. The models reconstructed from the SAXS data for PP-CC-AH-D, CC-AH-D, AH and PP-CC are colored black, red, green and blue respectively.


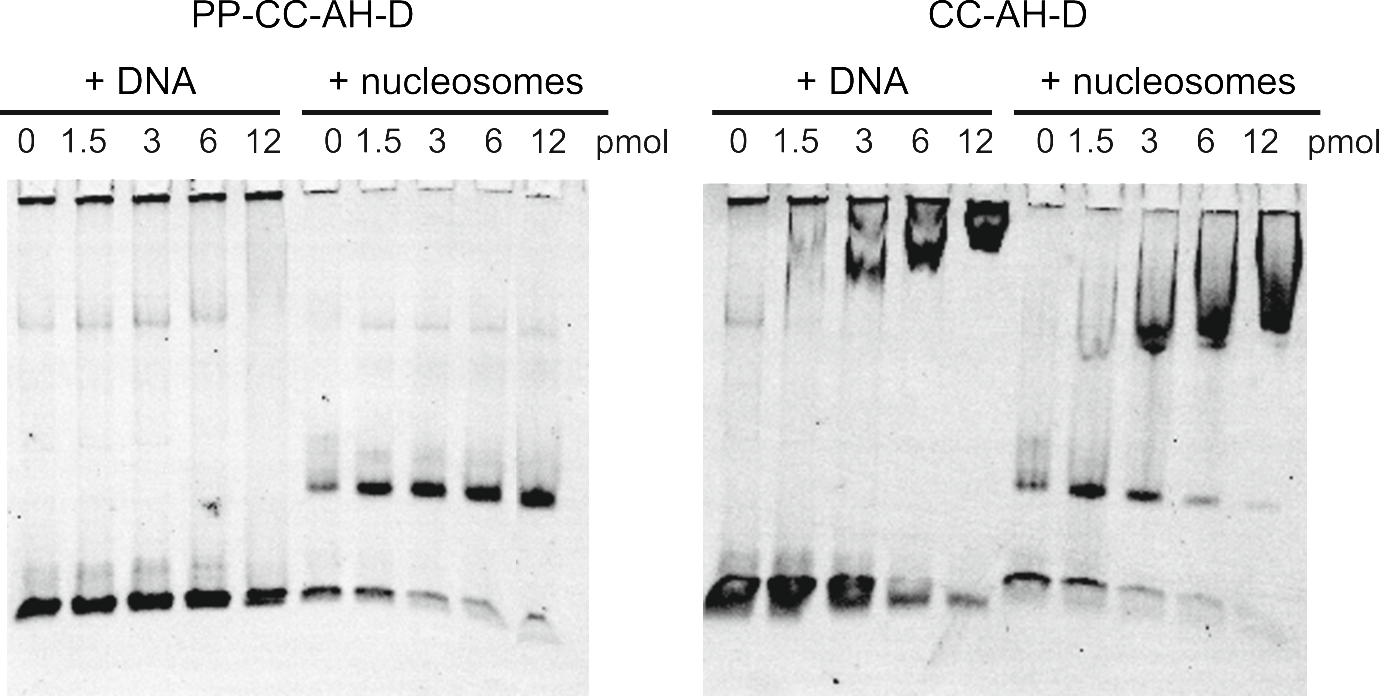


**Supplementary Figure 3. DNA and nucleosome binding gel shift of CHD4.**

A range of concentrations of the PP-CC-AH-D and CC-AH-D constructs of CHD4 were incubated with either 3 pmol of free DNA (Widom fragment, 0W0, 147 bp, Cy 3 labelled) or 3 pmol nucleosomes (human octamer on 0W0 DNA assembled, Cy 3 labelled) for 5 min at room temperature in 120 mM NaCl, 10 mM MgCl_2_, 50 mM Tris pH 8. Following the addition of 8 % sucrose to each mixture, samples were loaded onto a 5 % native polyacrylamide gel in 0.5x TBE for 1 hour at 150V and scanned with a phosphoimager.

| **Michaelis-Menten parameters** | **PP-CC-AH-D** |
| --- | --- |
| **Best-fit values** |  |
| V_max_ (pmol/min) | 16.87 |
| K_m_ (µM) | 1343 |
| **Std. Error** |  |
| V_max_ (pmol/min) | 0.7757 |
| K_m_ (µM) | 114.8 |
| **95% Confidence Intervals** |  |
| V_max_ (pmol/min) | 15.29 to 18.45 |
| K_m_ (µM) | 1109 to 1576 |
| **Goodness of Fit** |  |
| Degrees of Freedom | 33 |
| R square | 0.9912 |
| Absolute Sum of Squares | 3.119 |
| Sy.x | 0.3075 |
| Constraints |  |
| K_m_ | Km > 0.0 |
| **Number of points analyzed** | 35 |

**Supplementary Table 1. Enzymatic parameters and statistical variables for the ATPase activity of CHD4 in the presence of nucleosomes.**

The rates of ATP hydrolysis (ATP hydrolyzed per protein per minute) of CHD4 construct PP-CC-AH-D in the presence of an unmodified nucleosomal substrate were calculated at a range of ATP concentrations (0.007-2 µM final concentration) at 37 °C. The results of three independent experiments were included in the analysis. The enzymatic parameters K_m_ and V_max_ were calculated by nonlinear least-squares regression (GraphPad Prism; GraphPad Software Inc.) and standard statistical variables are presented. Sy.x represents the standard deviation of the vertical distances of each data point from the curve.
